# Ontogenetic Variation in the Thermal Biology of Yarrow's Spiny Lizard, *Sceloporus jarrovii*

**DOI:** 10.1371/journal.pone.0146904

**Published:** 2016-02-03

**Authors:** Anthony L. Gilbert, Matthew S. Lattanzio

**Affiliations:** 1 Department of Biological Sciences, Ohio University, Athens, Ohio, United States of America; 2 Department of Organismal and Environmental Biology, Christopher Newport University, Newport News, Virginia, United States of America; Universidad de la Republica, URUGUAY

## Abstract

Climate change is rapidly altering the way current species interact with their environment to satisfy life-history demands. In areas anticipated to experience extreme warming, rising temperatures are expected to diminish population growth, due either to environmental degradation, or the inability to tolerate novel temperature regimes. Determining how at risk ectotherms, and lizards in particular, are to changes in climate traditionally emphasizes the thermal ecology and thermal sensitivity of physiology of adult members of a population. In this study, we reveal ontogenetic differences in thermal physiological and ecological traits that have been used to anticipate how ectotherms will respond to climate change. We show that the thermal biological traits of juvenile Yarrow’s Spiny Lizards (*Sceloporus jarrovii*) differ from the published estimates of the same traits for adult lizards. Juvenile *S*. *jarrovii* differ in their optimal performance temperature, field field-active body temperature, and critical thermal temperatures compared to adult *S*. *jarrovii*. Within juvenile *S*. *jarrovii*, males and females exhibit differences in field-active body temperature and desiccation tolerance. Given the observed age- and sex-related variation in thermal physiology, we argue that not including physiological differences in thermal biology throughout ontogeny may lead to misinterpretation of patterns of ecological or evolutionary change due to climate warming. Further characterizing the potential for ontogenetic changes in thermal biology would be useful for a more precise and accurate estimation of the role of thermal physiology in mediating population persistence in warmer environments.

## Introduction

Understanding how thermal environments influence physiological and ecological traits of ectothermic taxa has become a common practice in predicting organismal responses to novel thermal regimes [1–3]. Rising ambient temperatures associated with climate change are limiting the time available for species’ activity, and the suitability of available habitats for meeting physiological demands, including growth and locomotor performance, highlighting a growing need to accurately predict species’ responses to these changes [4–11]. Often, the relationship between the thermal environment and the physiological and ecological traits of adult members of a species is used to infer their potential range of responses a species might exhibit. Although this approach has enhanced our understanding of the relationship between thermal biology and species persistence in warmer environments, it often overlooks consideration of earlier life history stages, and therefore potential ontogenetic variation in thermal physiological or ecological traits [12,13]. If such ontogenetic variation occurs, then distinct life stages (e.g. adults and juveniles) should differ in their susceptibility to climate change. Thus, models seeking to accurately predict species responses to climate change would then require examination of multiple relevant life history stages in a species.

Ontogenetic variation in thermal biology should be expected given the suites of age-related differences in biology in many vertebrate species [14–19]. Determining the degree of ontogenetic variation in thermal physiological and ecological characteristics may help resolve population-level variation in the severity of responses to changes in temperature. Focus on the propensity for selectable phenotypic variation to be produced during changes throughout an organism’s ontogeny has increased over the past few decades in areas outside of climate research [20,21]. For example, in lizards, studies have documented ontogenetic variation in locomotor performance [22], habitat use [23], and anti-predator behavior [24]. The potential for similar variation in thermal biological traits in this group remains understudied, (but see [13]), despite its potential to influence the ability of populations to respond to altered thermal niches. Variation in the physiological sensitivity of juvenile and adult animals to temperature may lead to differential survival between these age classes as ambient temperatures continue to rise. Consequently, population persistence depends not only on adult survival and reproductive success, but on recruitment to adulthood as well. Constriction of population growth rate due to juvenile mortality will reduce the effective size of a population and the pool of genetic variation [25], making it more difficult for species to cope with environmental change.

From an ecological standpoint, juveniles and adults might also exploit different environmental characteristics, possibly to reduce intraspecific competition [26,27], or because of differences in body size. The presence of ontogenetic variation in ecology is beneficial in terms of facilitating larger population sizes and allowing for a species to inhabit a broader range of environments [28]. For example, in *Anolis* lizards, differences in ecological traits throughout ontogeny often reflect the morphological and physiological differences between adults and juveniles [19,22,29], and ensure minimal competition between adults and juveniles for overlapping microenvironments. If thermal biological traits are fixed throughout development, then larger adults should outcompete juveniles for preferred thermal and ecological resources in an environment [30]. As a result, juveniles would exhibit reduced locomotor performance capacity and survival, hindering their recruitment into adulthood. Given these considerations, we expect adult and juvenile lizards to differ in their thermal biological traits [13].

Biophysical models of climate change for lizards support drastic increases in local and regional extinction events by the year 2100 due to altered thermal regimes [8,31]. Because lizards are dependent on external sources of temperature to regulate physiological activity and function [32–37], they are at high risk of extinction relative to other terrestrial vertebrate species. As with those for other ectothermic taxa, lizard biophysical models are predicated on the association between declines in population-level fitness of adult animals and rising environmental temperatures [8,38,39]. However, lack of consideration of earlier life stages in this process underestimates potential differences in the relative susceptibility of juvenile and adult lizards to climate change. In this study, we quantified aspects of the thermal biology of juvenile Yarrow’s spiny lizards, *Sceloporus jarrovii*. Adults of this species exhibit male-biased sexual size dimorphism (SSD), which has been attributed to a faster growth rate in males relative to females at earlier ontogenetic stages [40]. A previous study on the thermal biology of adult *S*. *jarrovii* from our study population found variation in these characteristics attributable to their SSD [41]. By quantifying the thermal traits of the juvenile members of this population, we sought to determine whether there are any ontogenetic differences in thermal biology in this species, as well as provide insight into the thermal sensitivity of this age class to climate change. Also, because adult *S*. *jarrovii* exhibit SSD, our findings should also provide insight into the potential physiological dynamics leading to adult differences in body size and thermal biology [41]. We test the predictions that (I) juvenile and adult *S*. *jarrovii* differ with respect to thermal ecology, (II) thermal tolerances, and their (III) thermal sensitivity of performance.

## Materials and Methods

### Study System, Morphology, and Housing of Animals

Animal care and experimental measures were approved by the Institutional Animal Care and Use Committee of Christopher Newport University (protocol 2015-8, to MSL). We collected 64 juvenile *S*. *jarrovii* (males, *n* = 33; females, *n* = 31) from Miller Canyon in the Huachuca Mountains of Cochise County, Arizona (elevation: 1920 m) during the first week of June 2015. This area is dominated by extensive rocky outcroppings and Ponderosa Pine. Adult *S*. *jarrovii* are most often found on large boulders or trees (*Pinus sp*.), whereas juvenile *S*. *jarrovii* are found utilizing smaller rocks (<25 cm diameter) or the ground (personal observation) at our site. *S*. *jarrovii* breeds in the fall, and females hold developing offspring overwinter, which is followed by parturition in late May/early June [42]. Juvenile *S*. *jarrovii* were unequivocally identified as such by their body size. Adult lizards in this population are >75mm in SVL [41], and juveniles are significantly smaller, thus the body size of the lizards we included in this study did not exceed 40.5mm. All animals were captured by hand or noose pole between the hours of 0800–1300, and we georeferenced all capture locations with a hand-held GPS (Garmin GPSMAP 62S, Garmin, USA). All lizards upon capture were transported in a climate-controlled vehicle to a laboratory facility at the Appleton-Whittell Research Ranch near Elgin, AZ. Lizards were housed in individual 18.4 cm L x 16.2 cm W x 6.7 cm H terraria and maintained on an ambient photoperiod set to match their external environment (14:10h light: dark). Each lizard was provided water *ad libitum*.

Adult *S*. *jarrovii* exhibit male-biased SSD [41,43], which has been associated with differences in thermal physiology. Although differences in body size are clear at adulthood, differences in body size may be evident at earlier stages in development. We measured snout-vent length (SVL), mass, head width, jaw length, and limb lengths to the nearest 0.1 mm using calipers. We also recorded the mass of each lizard to the nearest 0.1g using a Pesola^TM^ spring scale. Morphological measurements occurred in the laboratory following capture.

### Desiccation Tolerance

We estimated evaporative water loss (EWL) for all juvenile lizards, a phenotypic indicator of an individual organism’s ability to maintain water balance, an important trait in arid environments. EWL is a measure of the water lost due to the motion of air over integument, and is important for characterizing the risk of desiccation. Rates of EWL can be estimated to determine physiological costs of activity or movement [44] and can be influenced by body size [45,46], field activity levels [47], humidity, and ambient temperature [48,49]. We sought to estimate EWL as both a proxy for field activity and to link differences in thermal biology to EWL to provide insight into the physiological costs of sex-related differences in growth or thermal ecology. For example, if individuals are more active within their environment, the increased motion of air over their integument could lead to a concomitant rise in EWL rate.

Each lizard was placed into identical 12.25cm L x 9.2cm H x 6.0cm D terraria with a lid soldered with nine 0.5cm holes to allow for sufficient airflow into each container. The small size of these terraria is essential to minimize lizard movement during a trial. We measured EWL over a total of eight hours (1000 to 1800 h). We measured EWL at an ambient laboratory temperature of 30–32°C (<10% humidity), which is within the range of field-active temperatures for juvenile *S*. *jarrovii* (see Results). At the start of a trial and every two hours thereafter, we weighed each lizard in a container using an electronic balance sensitive to 0.001g. The rate of water lost between each two-hour interval was averaged and retained as our estimate of the rate of EWL for every individual. If a lizard defecated during a trial, the excrement was removed, weighed separately, and its mass excluded from the overall calculations.

### Thermal Ecology and Physiology

Our characterization of thermal ecology of *S*. *jarrovii* involved estimation of two traits that reflect their use of the surrounding thermal environment: field-active (T_*b*_) and substrate (T_*s*_) temperature. We recorded T_*b*_ and T_*s*_ in the field using a hand-held infrared thermometer (Raytek Raynger ST, Raytek, USA), sensitive to 0.2°C with a distance-to-spot ratio of 8:1. We measured field-active body temperatures immediately upon capture of a lizard by firmly pressing this thermometer against the lizards’ cloaca [41]. When infrared laser thermometers are firmly pressed against the animal’s cloaca, the surface temperature reading directly reflects their internal body temperature [50]. Previous studies have validated this approach for small-bodied lizards [51]. We then measured T_*s*_ on the substrate of the original position of the lizard using the IR thermometer (held ~10-cm above the substrate).

We used a laboratory thermal gradient (1.22m L x 0.31m W x 0.31m H) to estimate the preferred body temperatures (T_*pref*_) of all lizards [52]. Every lizard performed a thermal preference trial within 24hrs of capture. We ensured that lizards were not fed within 12hrs prior to testing their thermal preference because feeding has the potential to influence selected body temperatures in a thermal gradient [41,53]. A 2cm layer of sand served as a substrate in the arena. A light and heat source (60W light bulb) was suspended ~5cm above the arena at one end. At the opposite end, we placed an ice pack underneath the arena. Together, the cooling and heating sources generated a range of temperatures from 21 to 45°C. We adhered to the experimental protocol of Beal et al [41] for estimating the thermal preference of our lizards. In brief, we first measured the T_*b*_ of a focal lizard and placed it in the center of the gradient to initiate a trial. We then recorded its T_*b*_ after 30 minutes in the thermal gradient, and every 15 minutes thereafter, for a total of 90 min. The average of these five body temperature readings was retained as our estimate of that individual lizard’s T_*pref*_ [41].

Critical thermal limits represent the edges of physiological tolerance for ectotherms, and denote the range of temperatures at which physiological performance is possible [54]. Because critical thermal maximum (CT_*max*_) is often discussed as being a thermal physiological marker for susceptibility to climate warming, [55] we estimated this parameter for all juveniles (*n* = 64). Of those 64 individuals, we measured critical thermal minima (CT_*min*_) only for the subset of juveniles who were selected at random for thermal performance measures (*n* = 20), due to time constraints. To estimate CT_*max*_, we placed lizards in a heated rectangular enclosure using a 60W light bulb suspended 15cm above the enclosure. To estimate CT_*min*_, we cooled a separate enclosure with frozen gel pads to 0°C and placed lizards into the enclosure. These methods raise or lower a lizard’s body temperature by approximately 1°C per minute. We assessed lizards for loss of righting response every minute in both CT_*min*_ and CT_*max*_ trials by attempting to flip the animal on its back. We recorded the body temperature of the lizard at the point it lost its righting response as a measure of its CT_*min*_ or CT_*max*_ (to the nearest 0.2°C) using the same infrared thermometer as above.

We selected a subset of juveniles (*n* = 20; males = 10, females = 10) at random from our sample to estimate the thermal sensitivity of locomotor performance. Trials occurred within 48hrs of capture. We selected stamina as our physiological measure of locomotor performance, as stamina has been shown to influence various life history characteristics related to fitness in lizards, including anti-predator behavior, aggression, and territoriality [56,57]. We estimated stamina by chasing lizards around a circular racetrack with an outer diameter of 100cm, and an inner diameter of 60cm [58]. The racetrack was lined with 2cm of sand as a substrate. We suspended a 60W light bulb directly over the track to minimize the potential reduction in the body temperature of lizards throughout the duration of a stamina trial. We adjusted the height of the lamp based on the lizard’s target trial T_*b*_. We chased a lizard around the racetrack by lightly tapping on its tail. The time ran until loss of righting response was used as our estimate of stamina for each lizard [57,58].

We created thermal performance curves by obtaining a measure of stamina for each lizard at a series of specific body temperatures in a random order within its tolerance range [59]. We estimated stamina at 6 body temperatures: 33, 21, 30, 25, 37 and 28°C, and randomized the order in which individual lizards ran at each temperature. We used 60W light bulbs and ice packs to either raise or cool lizard T_*b*_ to the target temperature prior to a trial. Lizards were maintained at the target temperature for a minimum of 10 minutes prior to the initiation of a stamina trial. We raced each lizard once per body temperature, given the high repeatability of stamina trials [60] and as to not overstress juvenile lizards. We allotted >90 minutes between trials at different temperatures for lizards to recover, and all trials were conducted over a 48-hr period. In between trials, all lizards were returned to their original terraria and offered water *ad libitum*. Following the conclusion of these measurements, all lizards were returned to their original capture position.

### Data Analyses

We tested for morphological differences between the sexes using a multivariate analysis of variance. We then tested rates of EWL against mass, SVL and body condition (residuals of a mass-SVL regression) using analyses of covariance (ANCOVA) utilizing SVL, mass, or body condition as covariates, with sex as a fixed factor. We also tested for sex-related variation in all thermal characteristics, including T_*b*_, T_*s*_, T_*pref*_, CT_*min*_ and CT_*max*_, using ANCOVAs, using SVL as a covariate. In the models for T_*b*_ and T_*s*_ we also include time of day (h) as an additional covariate. We then tested for ontogenetic variation in thermal biological traits using the estimates of the same traits for adult *S*. *jarrovii* [41]. The thermal biological traits of adult *S*. *jarrovii* estimated in [41] were collected in an identical manner, during the same time of day and time of year in 2012.

We constructed thermal performance curves using general additive mixed models (GAMM)s in the “mgcv” v1.7–2.6 package in R [61]. Using a GAMM in order to construct a thermal performance curve is useful as GAMMs are robust enough to examine and accommodate nonlinearity of thermal performance data, while incorporating differences in curve shape and height that arise due to differences between or within grouping factors [41,62]. We incorporated body size (SVL) as a covariate while constructing our models to account for the effects of body size on performance [41]. Sex was incorporated as a fixed factor during the generation of TPCs. We first identified a null model of thermal performance as stamina fitted only to temperature (Model 0), and increased the complexity of this model by incorporating different covariates and factors. We first included body size (SVL) as a covariate (Model 1). We then incorporated sex as a factor, but did not include SVL as a covariate (Model 1a). Our final tested model incorporated both SVL as a covariate and sex as a factor (Model 2). We constructed these models using an autoregressive correlation structure in order to minimize the potential for within-individual performance effects on overall TPC shape and height, and a Gaussian error distribution. Models were scored using an Akaike Information Criterion (AIC). AIC values were compared across models, where a ΔAIC value >4 was used to determine if models represented different fits from one another [63]. We retained the model with the lower AIC score, or the simpler model if ΔAIC≤4, as the best-fit model to our data.

We compared juvenile thermal traits to adult traits measured by Beal et al. [41] using separate t-tests with thermal trait (either T_*b*_, T_*s*_, T_*pref*_, T_*opt*_ [thermal optimum of performance], CT_*min*_, or CT_*max*_) as the response variable and age (juvenile or adult) as the predictor. SVL was not included in these models because preliminary analyses showed no effect of this covariate on any thermal trait (all *P*>0.1). All analyses were performed in R v3.0.2 (R Development Core Team 2013). All means are presented ± 1 standard error (SE).

## Results

### Morphology

Although juvenile male *S*. *jarrovii* tended to be larger than females, we did not detect SSD in any morphological characteristic at this stage in development (*F*_1, 62_ = 1.19, *P* = 0.32, Table 1).

**Table 1:** Summary statistics of sample sizes, body size, mass and evaporative water loss of juvenile *Sceloporus jarrovii*. Means are presented with standard error in parentheses. Males and female juveniles displayed no dimorphism in morphology; therefore, we only report summary statistics of SVL and body mass.

Sex	*N*	Mean SVL (mm)	Mass (g)	Evaporative Water Loss (g/hr)
Male	33	33.95 (0.38)	1.34 (0.06)	0.0037 (0.0003)
Female	31	33.73 (0.57)	1.35 (0.05)	0.0027 (0.0002)

### Evaporative Water Loss

EWL rates were not influenced by SVL (*F*_1, 62_ = 1.11, *P* = 0.29), mass (*F*_1, 62_ = 2.08, *P* = 0.15), or body condition (*F*_1, 62_ = 0.001, *P* = 0.97). However, male lizards lost more water and had higher rates of EWL than females (*F*_2, 61_ = 3.843, *P* = 0.01, Table 1).

### Thermal Ecology

All thermal biological traits are summarized in Table 2. We found no difference in T_*s*_ between the sexes (*F*_1, 61_ = 0.255, *P* = 0.62), but lizards with greater SVLs had greater T_s_ than smaller lizards (*F*_1, 60_ = 6.136, *P* = 0.016). Male lizards however exhibited higher field-active T_*b*_’s than female lizards (*F*_1, 60_ = 12.29, *P*<0.001), but unlike T_*s*_, T_*b*_ did not vary with SVL (*F*_1,60_ = 2.124, *P* = 0.15).There was also no effect of time of day on either T_*b*_ or T_*s*_ (both *P*>0.09). Males and females selected similar body temperatures in the thermal gradient (*F*_1, 61_ = 0.26, *P* = 0.61). Juveniles selected higher T_*b*_’s in a laboratory gradient than their T_*opt*_ (*t* = -15.44, df = 37.6, *P*<0.001). Juveniles also were field active at T_*b*_’s higher than their T_*opt*_ (*t* = 5.97, df = 21.63, *P*<0.001).

**Table 2:** Summary of mean (standard error) thermal biological traits of juvenile *Sceloporus jarrovii* and published estimates of the same traits for adult *S*. *jarrovii* [41]. Sample sizes for adult *S*. *jarrovii* are *n* = 24 (males = 10 and females = 14) for traits except CT_*min*_ and CT_*max*_, where *n* = 10 (males = 5 and females = 5). Sample sizes for juvenile *S*. *jarrovii* are *n* = 64 (males = 33and females = 31) for traits except CT_*min*_ and T_*opt*_, where *n* = 20 (males = 10, females = 10).

	Juvenile			Adult
Thermal Trait (°C)	Male	Female	Overall	Overall
Field-Active T_*b*_	33.5(0.4)	31.5(0.4)	32.6(0.3)	32.8(0.4)
T_*s*_	32.6(0.8)	31.9(0.9)	32.3(0.6)	31.7(0.7)
T_*pref*_	33.0(0.2)	32.8(0.3)	32.9(0.2)	33.7(0.4)
CT_*min*_	16.5(0.2)	16.2(0.2)	16.4(0.1)	13.6(0.1)
CT_*max*_	40.3(0.4)	40.0(0.2)	40.2(0.1)	39.2(0.5)
T_*opt*_	30.3(0.3)	30.0(0.0)	30.2(0.2)	27.3(0.8)

### Critical Thermal Limits and Thermal Sensitivity of Performance

**Fig 1:**
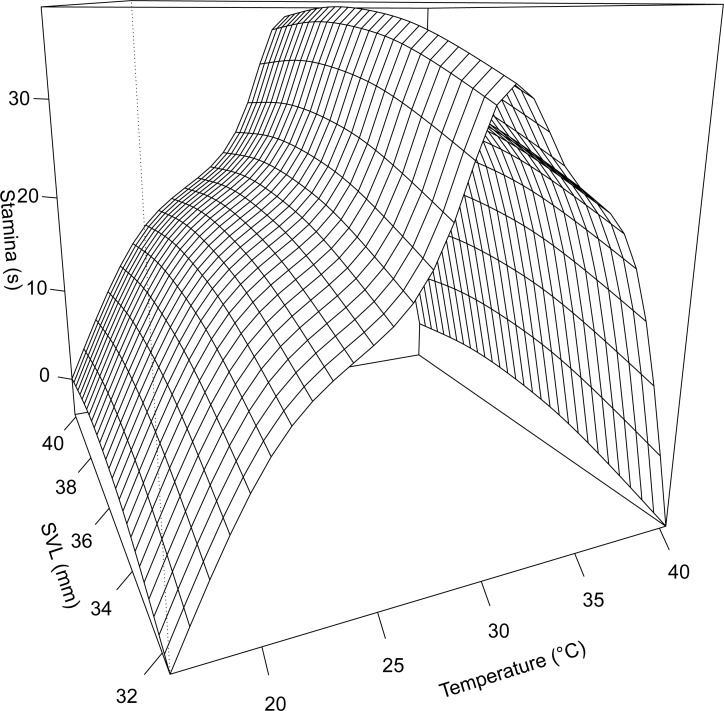
Generalized additive mixed models illustrating the thermal sensitivity of stamina performance capacity in juvenile *S*. *jarrovii*.

There was no difference in critical thermal limits between the sexes (CT_*max*_: *F*_1, 61_ = 0.14, *P* = 0.71, CT_*min*_: *F*_1, 17_ = 0.87, *P* = 0.37). Males and females displayed similar T_*opt*_’s (Table 2) and overlapping B95 (males: 29.6[0.1]– 31.4[0.3] °C; females: 29.4[0.2]– 31.1[0.1] °C) and B80 performance breadths (males: 28.4[0.1]– 33.6[0.3] °C; females: 28.0[0.3]– 34.5[0.5] °C). When we first examined the thermal performance curve for each sex, we detected little variation in the shape of the curve between the sexes. We therefore present a combined model of thermal performance incorporating both sexes (Fig 1). Our null model of thermal performance incorporated temperature and stamina for all individuals (Model 0: AIC = 847.26). Including SVL as covariate improved model fit (Model 1: AIC for model with SVL = 842.58, ΔAIC = -4.68), indicating an effect of body size on thermal performance. Excluding SVL and including only sex did not improve model fit (Model 1a: AIC = 844.07, ΔAIC = -3.19). Incorporating both SVL and sex into a model of thermal performance resulted in a better model fit compared to the null (Model 2: AIC = 840.06, ΔAIC = -7.2), but this model did not differ from a simpler model that only included SVL (ΔAIC = -2.42).

### Ontogenetic Differences in Thermal Traits

We detected no differences in field-active T_*b*_ or T_*s*_ by age (T_*b*_, *t* = 0.456, df = 47.714, *P* = 0.651; T_*s*_, *t* = -0.547, df = 57.664, *P* = 0.586). Adult *S*. *jarrovii* tended towards a higher T_*pref*_ (*t* = 2, df = 33.658, *P* = 0.053) and had a significantly lower CT_*min*_ (*t* = -15.045, df = 19.761, *P*<0.001) than juvenile lizards. In contrast, juvenile *S*. *jarrovii* tended to have a higher CT_*max*_ (*t* = -2.151, df = 9.87, *P* = 0.057) and higher T_*opt*_ (*t* = -3.723, df = 24.761, *P* = 0.001) than adult lizards.

## Discussion

Ontogenetic variation in thermal physiological traits may have important implications for the persistence of species in response to global climate change. Here, we characterized thermal physiological and thermal ecological traits of the juvenile age class of a population of high-elevation *S*. *jarrovii*. Our results, in combination with results of a previous study on adults of the same population [41], reveal age-specific variation in thermal physiological traits, supporting our predictions. Overall, juvenile male *S*. *jarrovii* are active at higher temperatures while in the field and display higher rates of EWL compared to juvenile females. Apart from these characteristics, there was no sex-related variation in any other thermal physiological trait. Juvenile *S*. *jarrovii* also were active in the field and preferred body temperatures in a thermal gradient warmer than their optimal performance temperature, highlighting an interesting mismatch in these thermal biological traits. Moreover, this variation in thermal biology will have implications for assessing the ability of *S*. *jarrovii* populations to persist in warmer environments.

Juvenile male *S*. *jarrovii* displayed increased rates of EWL and were field active at warmer temperatures than females of the same age class. Their field-active body temperatures were on average 3°C warmer than their optimal performance temperature, which can indicate several physiological possibilities. First, male *S*. *jarrovii* may not be active at temperatures for the optimization of stamina, and may instead be favoring a different type of performance, such as assimilation rate, growth, or sprint speed. For example, *S*. *jarrovii* are ambush predators, so their field-active body temperatures may reflect the thermal sensitivity of sprint speed or bite force, and not stamina. Second, the increased rates of EWL and a higher body temperature may be physiological consequences of higher activity patterns relative to females. Lastly, because adult *S*. *jarrovii* breed in the fall when temperatures are cooler, their optimal performance temperature may have evolved to exploit cooler temperatures during the autumnal reproductive season. However, we believe that the higher body temperatures of juvenile males serve to accommodate a faster growth rate relative to juvenile females.

Adult male *S*. *jarrovii* are on average 16% larger than females at our population [41], a consequence of faster juvenile male growth rates in this species [64]. Within *Sceloporus* lizards, a larger male body size at reproductive maturity is important for settling territorial disputes and may enhance reproductive success [65–67]. Juvenile males may be active at warmer temperatures to optimize their growth rate, and as a result need to be more active than females to procure the necessary energetic resources. This would lead to an increase in the rate of EWL. The feeding rates of juvenile lizards would need to be known to address this question, however. While we did not explicitly measure the thermal sensitivity of growth for *S*. *jarrovii*, EWL rates are temperature-dependent and thus higher body temperatures should also lead to higher rates of water loss in males in the wild. Thus although higher body temperatures may facilitate rapid growth rates, they also coincide with a physiological cost in terms of increased desiccation risk. This interaction between body temperature, desiccation risk, and growth rate would generate energetic and hydric demands that will be difficult to satisfy in altered thermal environments. These consequences will be exacerbated if rising temperatures force shifts in thermoregulation behavior, activity patterns, or thermal habitat use, that are maladaptive for growth [68–71]. Restriction of the time juveniles can be field active may result in increased energetic costs when they can be field active, due to a combination of higher body temperatures and EWL rates. Activity restriction can lead to a slower growth rate due to the inability to acquire sufficient energetic resources [72]. This trend may lead to selection favoring decreased body size at reproductive maturity, a common evolutionary response of ectotherms to hotter thermal regimes [73], demographic fluctuations [74], or both.

We also found sex-related differences in thermal physiological traits within juvenile lizards. In general, larger juveniles had higher field-active body temperatures and used warmer microhabitats than smaller animals. When controlling for the effects of body size, males displayed higher body temperatures while in the field than females, and both sexes preferred higher temperatures than needed for optimal stamina. The body temperature for males overlapped with their preferred temperature range in our thermal gradient. For males, activity at temperatures 3°C higher than their optimal performance temperature is associated with a 15.25% diminishment in overall locomotor performance capacity. In other words, juvenile males are field active at temperatures not beneficial for sustained periods of locomotor performance. We suggest that for male lizards, there may be a tradeoff in early ontogeny to favor growth or development at a faster rate at the cost of performance [68]. It may not be critical for juvenile lizards to maximize locomotor performance because many behaviors requiring sustained locomotion are associated with sexual maturity, including territory patrol, social displays, and aggressive competition for ecological resources [66]. Overall, it is more likely that the thermal ecology and physiology of juvenile lizards reflects the thermal sensitivity of growth and development rather than locomotor performance. In this case, fitness for juvenile lizards would not be as dependent on high levels of locomotor performance facilitating high territory holding potential or anti-predator behaviors. Rather, juvenile fitness would be dependent on the ability to grow to reach reproductive maturity at an appropriate body size for reproduction and territoriality. This trend may be exaggerated with SSD, because fitness for adult male *Sceloporus* lizards is linked with larger body sizes at reproductive maturity [62, 65], resulting in sex-related differences in thermal biology at early ontogenetic stages.

Several of the thermal traits we describe here reveal previously undocumented ontogenetic differences between juvenile *S*. *jarrovii* in our study and adult lizards from the same population and during the same season in 2012 [41] (Table 2). The traits of juvenile *S*. *jarrovii* that differ from adults include thermal preference (non-significant trend), critical thermal minimum and maximum, and thermal optimum of stamina performance. These differences highlight that ontogenetic variation in thermal biology can be significant within lizards. Larger ectotherms (including adult *S*. *jarrovii*) may be capable of tolerating lower temperatures than smaller-bodied ectotherms, likely reflecting physiological differences in cold tolerance throughout ontogeny. Likewise, tolerance of higher temperatures by smaller individuals should also be expected in this scenario. For juvenile *S*. *jarrovii*, tolerance of warmer temperatures may facilitate survival post-parturition because adult females give birth in early June at our study site, thereby forcing juveniles to satisfy energetic demands during the hottest time of year (June–August). The higher thermal optimum of juvenile lizards further supports the prediction that juvenile *S*. *jarrovii* are more tolerant of warmer environments than adults. Also, the general shape of our thermal performance curve differs from a thermal performance curve estimated for adult *S*. *jarrovii* (see Fig 3 of Beal et al. [41]). The differences in these performance curves, coupled with the fact that body size explained variation in both curves, support that the thermal-sensitivity of locomotor performance is body-size dependent in this species throughout ontogeny.

In the field, adult and juvenile lizards are active at similar body temperatures, which may increase competition for thermal microhabitat resources between these two age-classes. In support of that, both age classes were captured on microhabitats that overlapped in surface temperature. Minimization of intraspecific competition for these shared ecological resources throughout ontogeny would be advantageous for *S*. *jarrovii*, ultimately serving to favor a larger population size [30]. The fact that juveniles and adults differed in other thermal biological traits may serve to facilitate reduced competition for the same microhabitats despite similarities in mean body and substrate temperature between the age classes. A higher CT_*max*_ and T_*opt*_ would enable juveniles to exploit warmer substrates than adults. More data are needed however on the microhabitat selection and other aspects of thermal ecology (e.g. the distribution of operative temperatures describing the thermal quality of their microhabitat) of adult and juvenile lizards to better understand the role of resource competition, or other mechanisms, in driving the observed ontogenetic differences in thermal traits of *S*. *jarrovii*.

## Conclusions

The ability to delineate susceptibility between young versus old members of a threatened population should refine our understanding of the mechanisms by which changes in climate lead to population-level extinctions. The ontogenetic differences in *S*. *jarrovii* thermal biology we document in our study support the prediction that sensitivity to rising temperatures associated with climate change is also age-dependent in this species. Depending on how climate change manifests with respect to their thermal trait values, juveniles may be more susceptible to mortality as temperatures rise than adults (or vice-versa). A greater effect of warmer temperatures on the younger age classes of a population will be disadvantageous for sustaining a stable population size, particularly in species like *S*. *jarrovii* where selection favors fast growth rates in juveniles. Without an ecological or evolutionary response or a shift in geographic distribution, these populations are at risk of rapid extinction. Characterization of these potential age-specific vulnerabilities to rising temperatures will therefore be crucial in order to accurately describe long-term demographic responses of this, and similar, species to global climate change.

## Supporting Information

S1 Data Raw data used in manuscript. (XLSX)
